# Effect of acupuncture treatment in patients with mild to moderate atopic dermatitis: a randomized, participant- and assessor-blind sham-controlled trial

**DOI:** 10.1186/s12906-021-03306-1

**Published:** 2021-04-29

**Authors:** Jung Gun Park, Hyangsook Lee, Mijeong Yeom, Younbyoung Chae, Hi-Joon Park, Kyuseok Kim

**Affiliations:** 1 Department of Ophthalmology, Otorhinolaryngology and Dermatology of Korean Medicine, Graduate School of Korean Medicine, Kyung Hee University, Seoul, Republic of Korea; 2 Department of Korean Medical Science, Graduate School, Kyung Hee University, Seoul, Republic of Korea; 3 Acupuncture & Meridian Science Research Centre, College of Korean Medicine, Kyung Hee University, 26 Kyungheedae-ro, Dongdaemoon-gu, Seoul, 02447 Republic of Korea; 4 Department of Ophthalmology, Otorhinolaryngology and Dermatology of Korean Medicine, College of Korean Medicine, Kyung Hee University, 26 Kyungheedae-ro, Dongdaemoon-gu, Seoul, 02447 Republic of Korea

**Keywords:** Acupuncture, Atopic dermatitis, Clinical trial, SCORAD, Sham acupuncture

## Abstract

**Background:**

Atopic dermatitis (AD) is a chronic recurrent inflammatory skin disease that affects 1–3% of adults worldwide. Currently, it is not possible to completely cure AD; therefore, alternative treatments need to be developed to meet the patients’ needs. Here, based on our previous pilot study, we conducted the first confirmatory randomized controlled trial to evaluate the effect of acupuncture in patients with mild to moderate AD.

**Methods:**

A randomized, participant- and assessor-blinded, sham-controlled trial was designed with an intervention period twice-weekly for 4 weeks and a 4-week follow-up. We equally allocated 36 participants to the verum acupuncture (VA) and sham acupuncture (SA) groups. The main outcome measure was the change in SCORing Atopic Dermatitis index (SCORAD) score before and after treatment.

**Results:**

A total of 36 participants, aged 19 to 38 years, were enrolled, and 35 were included in the intention-to-treat analyses. The mean change in total SCORAD score differed significantly among the two groups at 4 weeks after randomization (*P* < .0001): the mean difference was − 11.83 (7.05) in the VA group and 0.45 (7.77) in the SA group. The mean SCORAD score substantially decreased 2-weeks after starting the acupuncture treatment and continued to improve for at least 4 weeks after the end of the treatment in the VA group compared to the SA group (each *P* < .0001). No serious adverse events were observed.

**Conclusions:**

Twice-weekly acupuncture treatment was effective in reducing AD symptoms in patients with mild to moderate AD without serious adverse events.

**Trial registration:**

Clinical Research Information Service Identifier: KCT0002796.

**Supplementary Information:**

The online version contains supplementary material available at 10.1186/s12906-021-03306-1.

## Background

Atopic dermatitis (AD) is a chronic, recurring inflammatory skin disease characterized by intense itching. AD mainly affects children; however, the prevalence in adults may range from 1 to 3% [1] to 17.6% [2], depending on the geographic region. Currently, the complete conventional cure of AD is not available; therefore, the AD management relies on relieving the symptoms including pruritus. Emollients, anti-inflammatory therapy (for example, topical corticosteroids and topical calcineurin inhibitors), and if needed, systemic therapy (for example, antihistamines, immunosuppressants, and corticosteroids) can be used [3]. However, occasionally patients’ symptoms are not fully ameliorated and long-term use of topical corticosteroids often causes adverse reactions [4, 5].

Due to the limitations of conventional therapy, the use of complementary and alternative therapies, such as acupuncture, is increasing [6, 7]. Functional magnetic resonance imaging studies have suggested that acupuncture alleviates allergen-induced itch in AD patients, which was not observed in the placebo or antihistamine group. In addition, they found that the putamen, a region implicated in motivation and habitual behavior underlying the urge to scratch, was associated with the anti-pruritic effects of acupuncture [8]. Effects of acupuncture on itch were also related to the connectivity between the mid-cingulate cortex and putamen in histamine-induced itch in healthy volunteers [9]. Although several studies [10–12] have suggested the clinical possibility of acupuncture treatment for AD, no randomized sham-controlled trial (RCT) has been performed [10, 13]. Therefore, we conducted a preliminary study on acupuncture treatment for AD to test its feasibility [14]. As a result, the study design was suggested as follows: using the SCORing Atopic Dermatitis index (SCORAD [total]), which is a useful tool to assess the severity of AD, as a primary outcome, with 36 participants undergoing a 4-week acupuncture period (twice weekly) and a 4-week follow-up. In addition, atopic diseases have been associated with functional gastrointestinal (GI) disorders recently [15], and our pilot study also suggested epigastric tenderness and dyspepsia seemed to be related to AD symptoms [14]. Thus, quantitative indicators of indigestion were also measured as a secondary outcome.

Based on the previous feasibility study, we conducted a randomized controlled clinical trial using a parallel design to assess the efficacy and safety of acupuncture treatment in mild to moderate AD. The present study is the first confirmatory RCT on the acupuncture treatment efficacy in patients with AD.

## Methods

### Ethics approval

This trial was approved by the Institutional Review Board at Kyung Hee University Korean Medicine Hospital and registered with the Clinical Research Information Service on April 13, 2018 (identifier: KCT0002796). The protocol of this clinical trial was in accordance with the Consolidated Standards of Reporting Trials [16] and Standards for Reporting Interventions in Clinical Trials of Acupuncture (STRICTA) guidelines and has been previously published [17]. We obtained written informed consent from all study participants before enrollment.

### Trial design

This was a randomized, participant- and outcome assessor-blind, randomized, sham-controlled trial to confirm the efficacy of acupuncture treatment in relieving symptoms of mild to moderate AD patients. This trial was conducted at Kyung Hee University Korean Medicine Hospital, Seoul, Korea, from January 2018 to August 2019. Posters regarding enrollment were placed on several notice boards across the Kyung Hee Medical Center, nearby schools, and local community centers, and advertisements were posted on websites.

### Participants

All data were collected at Kyung Hee University Korean Medicine Hospital. The inclusion criteria were as follows: 1) men and women aged 19–65 years who were able to read and write in the Korean language; 2) AD diagnosed according to the Hanifin and Rajka criteria [18]; 3) a score between 30 and 80 points on a 100-mm visual analog scale (VAS) of pruritus (VAS [Pruritus]) (0, no symptoms at all; 10, worst symptoms ever) [19]; 4) a score between 10 and 40 points on the objective SCORAD index (SCORAD [Objective]) [20]; 5) not using prescribed AD medications for a month; 6) agreed with the study protocol and signed written informed consent; and 7) not participating in other clinical studies in the last month.

The exclusion criteria were as follows: 1) severely fluctuating AD symptoms; 2) use of medications that may affect the symptoms of AD, or that the study was deemed inappropriate by the Korean Medicine Doctor (KMD); 3) secondary infection of AD lesions; 4) severe mental problems; 5) pregnancy, breastfeeding, or plans to have a baby during the study period; and 6) a medical history of a patient that the KMD deemed inadequate to participate in the trial.

### Randomization and blinding

Eligible participants (*N* = 36) who signed the written informed consent were assigned to either the verum acupuncture (VA) group (*n* = 18) or the sham acupuncture (SA) group (*n* = 18) in a 1:1 ratio. Independent statisticians generated a random number table using SAS 9.2 (PROC PLAN, SAS Institute Inc., Cary, North Carolina, USA) and sent it to the administrative staff at the Acupuncture and Meridian Science Research Center (AMSRC) at Kyung Hee University. Random numbers and group assignments were sealed in sequentially numbered opaque envelopes and kept in the AMSRC.

Participants and outcome assessors were blinded to the group allocations, except the KMD, who conducted acupuncture treatments because of the nature of the treatment. The results were evaluated and analyzed by independent researchers who did not conduct the intervention. To ensure that the participants were blinded to their allocation, a blinding screen and Park sham device (AcuPrime Co. Ltd., Exeter, UK) were used during the acupuncture procedure.

### Interventions

Acupuncture treatments were based on the revised STRICTA 2010 recommendations [21] (Supplementary Table 1) and a predefined protocol [17]. All acupuncture treatments were conducted by one KMD with more than 2 years of clinical experience in Korean Medicine dermatology who had undergone more than 10 h of education about this trial protocol.

Participants received acupuncture or sham-acupuncture treatment (after group allocation) twice a week for 4 weeks (total 8 times). At week 8 (that is, 4-weeks after the treatment ended), the participants underwent a final examination. No other intervention was performed during the last visit.

#### VA group (treatment group)

The VA group received acupuncture treatments twice a week for a total of 4 weeks. Disposable sterile stainless needles (0.25 × 40 mm; Dongbang Acupuncture Inc., Bundang, Seongnam, Korea) were used. Six fixed acupoints (LI11, ST36, and PC6, bilaterally) were used in all the VA group participants. Additionally, up to 10 acupoints (ST43, GB41, LI2, TE3, SI3, TE6, SI2, BL66, LR3, and SP3) were used depending on each participant’s symptoms. Acupoints were selected based on traditional meridian theory and consensus by expert clinicians.

Acupuncture needles were inserted 5–30 mm deep, depending on the locations of acupoints, and the manipulation technique was performed at each acupoint until the “de qi” sensation was induced. The acupuncture needles were retained for 15 min. After removal of the acupuncture needles, intradermal acupuncture using 1.5 mm press tack needles (Haeng Lim Seo Won Medical Co., Korea) was applied at three acupoints (LI11 bilaterally, auricular Shenmen contralaterally).

#### SA group (control group)

The SA group received sham acupuncture treatment twice a week for a total of 4 weeks, similar to the VA group. Non-penetrating SA (Park sham acupuncture needles, AcuPrime Co., Ltd., Exeter, UK) was used. Six fixed control points (1 cm proximal and 1 cm medial to LI11, 1 cm proximal and 1 cm lateral to ST36, and 1–2 cm proximal and 1 cm medial to LI7, bilaterally) were used in the SA group. SA needles did not penetrate the skin, and no manipulation technique was used. The SA needles were retained for 15 min. After removal of the needles, ring-sham press tack needles that did not penetrate the skin were applied at three control points (1 cm proximal, and 1 cm medial to LI11 bilaterally, and the finger point in the ear contralaterally). All other procedures were the same as those used in the VA group.

### Outcomes

Outcome measures were determined based on a pilot study [14]. The primary outcome was the change in SCORAD (Total) score before and after treatment (baseline to week 4). Changes in SCORAD (Total) scores were calculated by subtracting the baseline SCORAD (Total) from week-4 SCORAD (Total) score. Therefore, the negative value of changes in the SCORAD (Total) score indicated improvement of symptoms.

Secondary outcomes were as follows: (1) baseline to week 4 changes in AD symptoms and quality of life (QoL); (2) changes between baseline and weeks 2, 4, and 8 in AD symptoms and QoL; and (3) the severity of dyspeptic symptoms.

The evaluation indicators can be divided into four categories: (1) measures of the severity of AD symptoms: SCORAD, Eczema Area and Severity Index (EASI), and Patient-Oriented Eczema Measure (POEM); (2) a measure of the QoL affected by AD: Dermatology Life Quality Index (DLQI); (3) measures of the severity of dyspeptic symptoms: Nepean Dyspepsia Index Korean version (NDI-K), VAS for dyspepsia (VAS [Dyspepsia]), Adequate Relief of gastrointestinal (GI) discomfort, and abdominal pressure pain threshold (APPT) using an algometer developed in the Korea Institute of Oriental Medicine; and (4) others: credibility test, blinding test, and assessment of adverse effects. SCORAD assesses the severity of AD by its extent, intensity, and subjective symptoms, especially pruritus and insomnia [20]. EASI is an effective tool for objectively evaluating the severity of eczema [22]. POEM is a validated tool for evaluating the severity of eczema in patients [23]. The DLQI is used to assess the QoL of patients with dermatologic symptoms [24].

In this trial, quantitative indicators of indigestion were added because epigastric tenderness and dyspepsia seemed to be related to AD symptoms in our pilot study [14]. NDI is a reliable scale for evaluating dyspepsia symptoms and the related QoL [25], whereas AR assesses the overall improvement in GI discomfort. APPT was measured to quantitatively assess abdominal symptoms using a new algometer developed at the Korea Institute of Oriental Medicine [26]. The detailed procedure has been described previously [17]. The credibility test was used to evaluate the reliability and expectations of the acupuncture treatment [27]. To assess blinding, participants were asked to which group they belonged (the VA group or SA group) at the end of the study [28].

The participants completed all questionnaires, and a researcher, blinded to the group allocation, performed the objective assessments.

The SCORAD, EASI, POEM, DLQI, and VAS (Dyspepsia) were measured weekly during the treatment period (baseline to week 4) and on the last visit (week 8). The NDI-K was conducted at baseline, week 4, and at the last visit (week 8). The APPT was measured at baseline and weeks 2 and 4. Adequate relief of GI discomfort was measured at weeks 3, 4, and 8. The credibility test was performed at baseline and week 4. The blinding test was performed at week 4. During each visit, the patients were asked to report any experienced adverse effects.

### Sample size

The sample size was calculated based on the pilot study [14]. Changes in SCORAD (Total) scores before and after treatment (baseline to week 4) were used as ground data. The number of samples was calculated using G*Power (G*Power 3.1.9.2 for Windows 10, URL: http://www.gpower.hhu.de). As a result, we needed 18 patients in each group, bringing the total sample size to 36 (effect size, d = 1.078, critical t = 2.048, Df = 28, noncentrality parameter δ = 2.951, actual power = 0.813, using independent t-test, α-level = 0.05, power = 80%, and dropout rate = 20%).

### Statistical analysis

The baseline characteristics and clinical outcomes described were based on the intention-to-treat (ITT) population, which included participants who had at least one treatment and one primary outcome measure. The mean (± standard deviation [SD]) was calculated for each continuous variable, and binary variables were presented as numbers (%). The missing data of participants who dropped out were replaced by the last observation carried forward method. If the data were normally distributed, we used the independent t-test to detect differences between the two groups. If the data distribution was not normal, we used the Wilcoxon rank-sum test. The chi-squared test or Fisher’s exact test was used for categorical variables. To avoid possible errors related to the initial value, analysis of covariance (ANCOVA) was used to determine the difference between post values of groups after adjusted pre-values. We compared changes in baseline up to 8 weeks through the repeated measures analysis of variance (RM-ANOVA), which estimated the least-squares mean by time and group. The *p*-value was set as 0.05, and the SAS 9.4 program (SAS Institute Inc., Cary, NC) was used for statistical analyses.

## Results

### Study participants and baseline characteristics

**Fig. 1:**
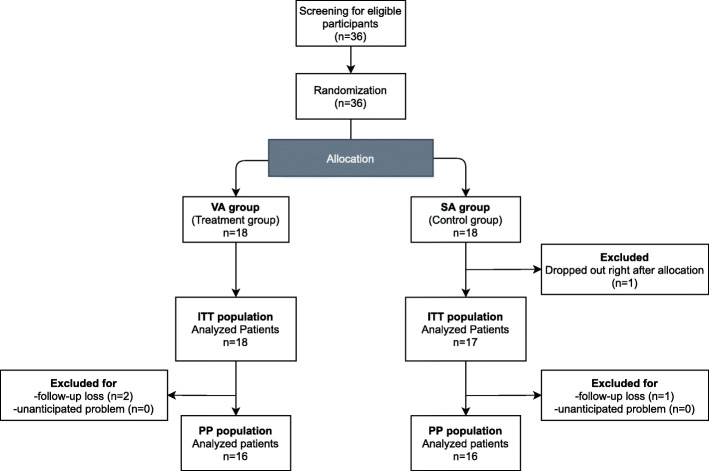
Flow chart of double-blinded, randomized sham-controlled trial

Initially, 36 participants were enrolled in the study. One participant in the SA group dropped out immediately after group allocation for personal reasons and was excluded from the ITT analysis. A total of 35 patients (mean [SD] age 23.78 [5.02] years; 21 [70%] female) participated in the study. Two participants in the VA group and one in the SA group were excluded from per-protocol (PP) analysis due to follow-up loss (Fig. 1). Similar results were obtained from the ITT and PP analyses, and the results for the ITT analysis are shown in Table 1. There were no significant differences in the baseline characteristics between the VA and SA groups.

**Table 1:** Baseline characteristics of the participants

Characteristic	VA (***n*** = 18)	SA (***n*** = 17)	***P***-value
Gender (Male/Female)	7/11 (38.89%)	7/10 (41.18%)	0.890
Age (Years)	24.67 (5.27)	22.82 (4.71)	0.332
Body mass index (kg/m^2^)	23.03 (3.86)	22.91 (3.39)	1.000
SCORAD (Total)	34.38 (8.09)	34.90 (10.64)	0.922
SCORAD (Objective)	24.90 (7.34)	25.53 (9.15)	0.823
VAS (Pruritus)	5.72 (1.09)	6.04 (0.92)	0.358
VAS (Insomnia)	3.76 (2.88)	3.33 (2.36)	0.707
EASI	5.16 (3.75)	5.83 (5.58)	0.987
POEM	12.39 (4.47)	14.18 (5.77)	0.312
DLQI	8.83 (4.72)	10.24 (6.21)	0.577
VAS (Dyspepsia)	4.02 (2.83)	3.32 (2.69)	0.456
NDI-K	19.72 (19.74)	22.06 (20.31)	0.694
Credibility test	18.00 (1.85)	17.41 (2.65)	0.592

*P* > 0.05 for all comparisons. Values are expressed as mean (standard deviation) or count (%). Continuous data were analyzed using independent t-test. If data were not normally distributed, then wilcoxon rank sum test was used. Bivariate data were analyzed using chi-squared test

*VA* Verum Acupuncture, *SA* Sham Acupuncture, *SCORAD* SCORing Atopic Dermatitis index, *VAS* Visual Analog Scale, *EASI* Eczema Area and Severity Index, *POEM* Patient-Oriented Eczema Measure, *DLQI* Dermatology Life Quality Index, *NDI-K* Nepean Dyspepsia Index-Korean version

### Primary endpoints

**Fig. 2:**
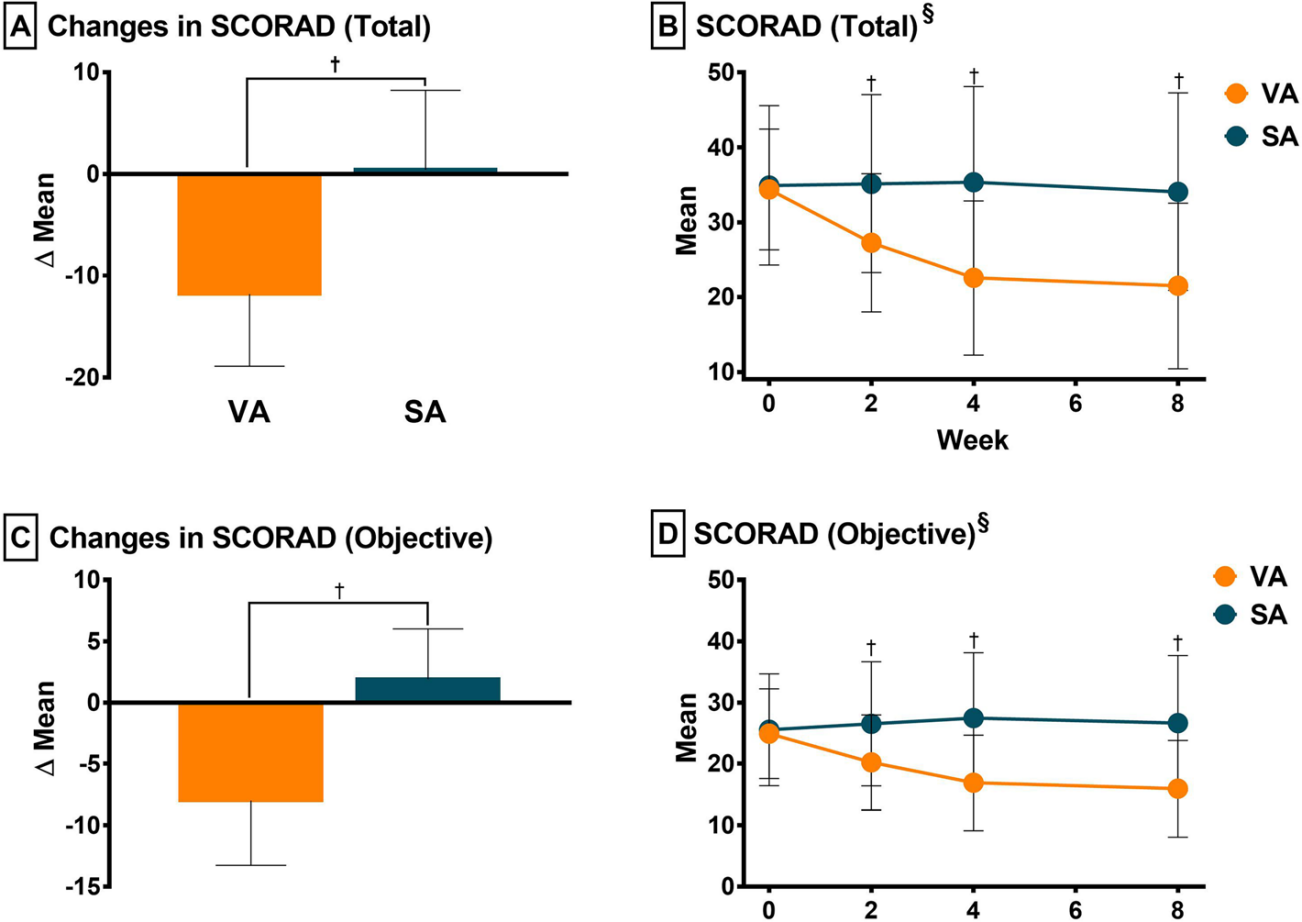
Changes in SCORAD total (**a, b**) and objective (**c, d**) scores. **a** was analyzed using the independent t-test. **c** was analyzed using Wilcoxon rank-sum test. **b, d** were analyzed using ANCOVA to compare the SCORAD scores between groups at weeks 2, 4, and 8, respectively. RM-ANOVA was also used to analyze changes of the SCORAD scores between groups over time. ^§^Time and group interaction was found. ^†^ *p* < 0.01. SCORAD, SCORing Atopic Dermatitis index; ANCOVA, analysis of covariance; RM-ANOVA, repeated measures analysis of variance

The mean (SD) change in the SCORAD (Total) score differed significantly between the VA and SA groups at 4 weeks after randomization (*p* < .0001): the mean difference (SD) was − 11.83 (7.05) in the VA group and 0.45 (7.77) in the SA group (Fig. 2a).

### Secondary end points

#### Changes in the SCORAD score over time

ANCOVA was conducted to analyze changes in the SCORAD score between the two groups at baseline and weeks 2, 4, and 8. The baseline scores of the participants were used as covariates to control for any potential pre-existing differences. Differences between the VA and SA were seen as early as week 2, with further improvement to week 4. This improvement lasted for at least 4 weeks after the completion of the final acupuncture treatment (each *p* < .0001). Using RM-ANOVA, time and group interactions were found between the two groups (Fig. 2b).

#### Changes in objective outcomes associated with AD

There were significant differences between the VA and SA groups concerning changes in SCORAD (Objective) scores (*p* < 0.0001, Fig. 2c) and EASI (*p* = 0.0025) before and after treatment (Table 2). ANCOVA analysis revealed significant differences between VA and SA groups at weeks 2, 4, and 8 with regard to SCORAD (Objective, Fig. 2d) and EASI scores, respectively.

**Table 2:** A comparison of changes in AD symptoms between baseline and week 4 and periodic analysis of AD symptoms

Variables	Verum Acupuncture (***n*** = 18)	Sham Acupuncture (***n*** = 17)	Difference between groups after adjusted baseline value (95% CI)	***P*** value^a^
**Total SCORAD**^**b**^				
LS mean (SE) at baseline	34.38 (2.30)	34.90 (2.37)	–	–
LS mean (SE) at week 2	27.25 (2.60)	35.12 (2.67)	7.37 (3.46, 11.29)	0.0036
LS mean (SE) at week 4	22.55 (2.83)	35.35 (2.91)	12.31 (7.14, 17.48)	<.0001
LS mean (SE) After treatment, at week 8	21.50 (2.97)	34.06 (3.06)	12.06 (6.36, 17.77)	0.0006
**Objective SCORAD**^**b**^				
LS mean (SE) at baseline	24.90 (2.02)	25.53 (2.08)	–	–
LS mean (SE) at week 2	20.22 (2.20)	26.50 (2.27)	5.66 (3.02, 8.29)	0.0006
LS mean (SE) at week 4	16.90 (2.27)	27.45 (2.34)	9.94 (6.64, 13.25)	<.0001
LS mean (SE) After treatment, at week 8	15.94 (2.34)	26.62 (2.40)	10.12 (5.82, 14.42)	<.0001
**EASI**^**b**^				
LS mean (SE) at baseline	5.16 (1.16)	5.83 (1.19)	–	–
LS mean (SE) at week 2	4.47 (1.26)	5.97 (1.29)	0.78 (0.02, 1.55)	0.2754
LS mean (SE) at week 4	3.44 (1.22)	6.49 (1.26)	2.40 (0.99, 3.81)	0.0090
LS mean (SE) After treatment, at week 8	3.29 (1.29)	6.13 (1.33)	2.15 (0.74, 3.57)	0.0246
**POEM**				
LS mean (SE) at baseline	12.39 (1.26)	14.18 (1.29)	–	–
LS mean (SE) at week 2	9.44 (1.46)	12.35 (1.51)	1.36(−1.48, 4.20)	> 0.9999
LS mean (SE) at week 4	7.72 (1.53)	11.65 (1.57)	2.37(−0.73, 5.48)	0.7758
LS mean (SE) After treatment, at week 8	7.78 (1.70)	11.76 (1.75)	2.02(− 0.82, 4.85)	0.9432
**DLQI**				
LS mean (SE) at baseline	8.83 (1.34)	10.24 (1.38)	–	–
LS mean (SE) at week 2	6.28 (1.00)	6.76 (1.02)	−0.26(−2.25, 1.72)	> 0.9999
LS mean (SE) at week 4	5.06 (0.95)	5.76 (0.98)	0.23(−2.18, 2.64)	> 0.9999
LS mean (SE) After treatment, at week 8	4.72 (1.03)	5.47 (1.06)	0.10(−2.27, 2.48)	> 0.9999

*Abbreviations*: *SCORAD* Scoring Atopic Dermatitis (range, 0 [clear] to 100 [very severe]), *EASI* Eczema Area and Severity Index, *POEM* Patient-Oriented Eczema Measure (range, 0 [clear] to 28 [very severe]), *DLQI* Dermatology Life Quality Index (range. 0 [no effect of skin disease on quality of life] to 30 [maximum effect on quality of life])

^a^ From an analysis of covariance (ANCOVA) with a factor of treatment group and corresponding baseline value as the covariate. The *p*-value is corrected by multiple comparisons

^b^ This shows a significant difference between groups through repeated measures ANOVA (RM ANOVA). In addition, the LS mean is the least squares mean estimated by RM ANOVA

#### Changes in subjective outcomes associate with AD

Statistically significant improvements were observed within each group over time, but no significant differences between VA and SA groups in terms of changes in VAS (Pruritus), VAS (Insomnia), POEM, and DLQI scores before and after treatment (Table 2) were present.

##### Changes in dyspeptic symptoms

Based on the ANCOVA analysis, using the baseline score of participants as a covariate, statistically significant differences were observed between the VA and SA groups in VAS (Dyspepsia) (*p* = 0.0135) and NDI-K (*p* = 0.0119) scores at week 4. In the adequate relief of gastric discomfort, there was a significant difference between the VA and SA groups, but no significant difference was identified within each group (Supplementary Fig. 1). There was no significant difference between the VA and SA groups in the change in APPT scores between weeks 0 and 4.

Using Spearman’s rank correlations, there were statistically significant correlations between SCORAD (Total) and VAS (Dyspepsia) scores at baseline (r_s_ = .38, *p* = 0.022) and at week 4 (r_s_ = .514, *p* = 0.003).

##### Credibility and blinding tests

There was no significant difference in the analysis of the blinding test between the VA and SA groups, as assessed by the chi-square test (*p* = 0.0719) (Supplementary Table 2). The credibility of the acupuncture treatment was similar in both the VA and SA groups over time (baseline, 4, and 8 weeks). The degree of credibility ranged from 16.8 to 18.0 of 24 points in both groups (Supplementary Table 3).

##### Safety assessments

Four adverse events were observed during the study: two instances of dyspepsia and one each of heartburn and numbness of the left forearm. There might be a causal relationship between acupuncture treatment and numbness of the arm, but the case was not severe. Other cases were not included in this study. No serious adverse events occurred during the study period.

## Discussion

Twice-weekly acupuncture treatment for 4 weeks improved AD symptoms in adults with mild to moderate AD compared with sham acupuncture. The VA group showed statistically significant improvement in the primary endpoint (mean change in the SCORAD [total] score from baseline to week 4) compared to the sham acupuncture group.

SCORAD (Total), SCORAD (Objective), and EASI scores are the best objective instruments to assess the clinical signs of AD [29]. In this study, we observed the statistically significant differences in all three instruments between the VA and SA groups, indicating that acupuncture can induce objective improvements in AD symptoms. Considering the minimal clinically important difference for adult patients is 8.7 points for SCORAD (Total) [30], the improvement of 11.83 induced by acupuncture treatment may suggest that VA is not only superior to SA but also resulted in significant clinical improvements in AD patients.

The mean SCORAD (Total), SCORAD (Objective), and EASI scores were substantially decreased 2-weeks after starting the acupuncture treatment, and continued to improve for at least 4-weeks after the final treatment in the VA group as compared to the SA group. It is interesting that the objective AD symptoms, improved by acupuncture treatment were maintained for at least 4 weeks, and it is meaningful if these effects can last for longer term in further studies.

In contrast, a significant difference between the VA and SA groups was not found in the subjective outcomes such as VAS (Pruritus), VAS (Insomnia), POEM, and DLQI score. However, all subjective indices significantly improved in both groups after treatment compared to before treatment. We speculate that this might be due to the inherent non-specific effects of acupuncture. It is well known that both specific and non-specific effects contribute to the effects of acupuncture, and the non-specific effects of acupuncture come from placebo, expectancy, Hawthorne, and Pygmalion effects [31]. It has been reported that placebo effects differ in the subjective or objective outcome, which may explain the discrepancies in the significance between subjective and objective AD symptoms in this study: the placebo was more effective than no treatment in trials assessing subjective outcomes, while the placebo did not differ from no treatment in objective outcomes [32].

Recently, atopic diseases have been reported to be associated with functional GI disorders such as functional dyspepsia and irritable bowel syndrome [15]. Moreover, adults with AD had higher odds of developing gastroenteritis than those without AD [33, 34]. In this study, we found the GI function such as dyspepsia and gastric discomfort correlated with AD symptoms (SCORAD). In addition, acupuncture treatment resulted in relief of dyspeptic symptoms and improvement of objective AD symptoms. It has been shown that an abnormality in the autonomic nervous system can be associated with immunological and neurophysiological imbalance [14, 35], which might contribute to GI dysfunction in patients with AD. Previously, we reported that participants with higher parasympathetic activation induced by acupuncture treatment produced a higher reduction of itch [9], and it was also reported that acupuncture could decrease systemic inflammation via vagal modulation [36]. These findings might partially explain how acupuncture alleviates AD and comorbid gastric discomfort. Further studies are warranted to investigate the relationship between AD and gastrointestinal function and the mechanism underlying acupuncture treatment affecting both symptoms simultaneously.

### The main strength of this study

To the best of our knowledge, this is the first study to assess the efficacy of acupuncture in AD patients with a calculated sample size in a pilot study. The treatment protocol was based on the best practice of acupuncture and was developed by expert clinicians; thus, it has broad generalizability to the practice of acupuncture. We also observed that acupuncture could alleviate dyspeptic and objective AD symptoms, which might be beneficial for AD patients with GI discomfort.

### Limitations

This study has several limitations. These include the applicability of our results to other population groups, such as children or older patients. In addition, a short treatment period and follow-up should also be considered as limitations of this clinical trial. This study only included patients with mild to moderate AD, so the effects of acupuncture in patients with severe AD need to be further explored. Further trials should consider all these issues to assess the effects of acupuncture.

## Conclusions

Throughout this study, we found that twice-weekly acupuncture treatment for 4 weeks improved AD symptoms in the VA group compared to the SA group. The treatment seemed to be effective from the second week onwards, and the effectiveness was maintained even after the discontinuation of the acupuncture treatment.

## Supplementary Information

**Additional file 1: Supplementary Table 1.** Acupuncture treatment details based on the checklist for STRICTA 2010. **Supplementary Table 2.** Analysis of the blind test. **Supplementary Table 3.** Analysis of the credibility test. **Supplementary Fig. 1.** Periodic analysis of dyspeptic symptoms.

## Data Availability

The datasets used and/or analyzed in this article are available from the corresponding author on reasonable request.

## Abbreviations

AD: Atopic dermatitis
AMSRC: Acupuncture and Meridian Science Research Center
ANCOVA: Analysis of covariance
APPT: Adequate Relief of gastrointestinal discomfort, and abdominal pressure pain threshold
DLQI: Dermatology Life Quality Index
EASI: Eczema Area and Severity Index
GI: Gastrointestinal
ITT: Intention-to-treat
NDI-K: Nepean Dyspepsia Index Korean version
POEM: Patient-Oriented Eczema Measure
PP: Per-protocol
QoL: Quality of life
RCT: Randomized sham-controlled trial
RM-ANOVA: Repeated measures analysis of variance
SA: Sham acupuncture
SCORAD: SCORing Atopic Dermatitis index
SD: Standard deviation
VA: Verum acupuncture
VAS: Visual analog scale
