# Supplementary material for: Effect of acupuncture treatment in patients with mild to moderate atopic dermatitis: a randomized, participant- and assessor-blind sham-controlled trial
Source: BMC Complement Med Ther. 2021 Apr 29;21:132. doi: 10.1186/s12906-021-03306-1 (PMC8082608; doi:10.1186/s12906-021-03306-1)
Supplement: Supplementary file 1 — Additional file 1: Supplementary Table 1. Acupuncture treatment details based on the checklist for STRICTA 2010. Supplementary Table 2. Analysis of the blind test. Supplementary Table 3. Analysis of the credibility test. Supplementary Fig. 1. Periodic analysis of dyspeptic symptoms. [file 12906_2021_3306_MOESM1_ESM.docx]

Supplementary table 1. Acupuncture treatment details based on the checklist for STRICTA 2010

| Item | Detail |
| --- | --- |
| 1) Acupuncture Rationale | 1a) Style of acupuncture  i) MA and IA using PTN based on traditional meridian theory  1b) Reasoning for treatment provided, based on historical context, literature sources, and/or consensus methods, with references where appropriate  i) Partially individualized MA treatment and IA treatments based on the traditional meridian theory, clinical experience, and consensus by the experts in acupuncture and AD  1c) Extent to which treatment was varied  MA: Partially individualized, that is, 6 fixed points plus optional points according to individual symptoms  IA: Fixed treatment |
| 2) Details of Needling | 2a) Number of needle insertions per subject per session (mean and range where relevant)  MA: 6 to 19 body acupoints per participant per session  IA: 2 body acupoints and 1 auricular acupoint per participant per session  2b) Names (or location if no standard name) of points used (uni/bilateral)  MA:  i) fixed points: PC6, LI11, ST36 bilaterally  ii) optional points: ST43, GB11, LI2, TE3, TE6, SI2, SI3, BL66, LR3, SP3 contralaterally (GB41, TE3, SI3 can be applied bilaterally according to the signs or symptoms of the patient)  IA: LI11 bilaterally and auricular shenmen contralaterally  2c) Depth of insertion, based on a specified unit of measurement or on a particular tissue level  MA: From 5 to 30 mm, perpendicular to skin surface  IA: 1.5 mm, perpendicular to skin surface  2d) Response sought (e.g., de qi or muscle twitch response)  MA: “de qi” sensation  IA: None  2e) Needle stimulation (e.g., manual, electrical)  MA: Manual stimulation, needle rotation with thumb and index fingers for the first 10-15 seconds  IA: Participants will be educated to press PTNS at LI11 for more than 3 minutes when they feel severe itching  2f) Needle retention time  MA: 15 minutes  IA: 1-2 days until PTN falls off  2g) Needle type (diameter, length, and manufacturer or material)  MA: A sterilized stainless steel needle (0.25 x 40 mm, Dongbang Acupuncture Inc., Bundang, Seongnam, Korea)  IA: A hypoallergic PTN (1.5 mm, 10 x 10 mm adhesive tape, Haeng Lim Seo Won Medical Co., Korea) |
| 3) Treatment Regimen | 3a) Number of treatment sessions  i) 8 sessions  3b) Frequency and duration of treatment sessions  i) Twice a week for 4 weeks, 15 minutes for each session |
| 4) Other Components of Treatment | 4a) Details of other interventions administered to the acupuncture group (e.g. moxibustion, cupping, herbs, exercises, lifestyle advice)  i) Lifestyle advice will be given to all participants  ii) Any other interventions will be prohibited during study period  4b) Settings and context of treatment, including instructions to practitioner and information and explanations to patients  i) Participants will be informed about acupuncture treatment in the study as follows: "In this study, you will be randomly allocated to VA group or SA group. Acupoints will be selected based on traditional Korean medicine textbook and AD-related reports. Also, additional acupoints can be used according to individual conditions, if needed." |
| 5) Practitioner Background | 5) Description of participating acupuncturists (qualification or professional affiliation, years in acupuncture practice, other relevant experience)  i) KMD who has clinical experience in Korean Medicine dermatology more than 2 years. The practitioner KMD will have undergone more than 10 hours of training and simulation workshop to ensure that he is able to provide identical acupuncture treatment in accordance with a pre-defined protocol. |
| 6) Control and Comparator Interventions | 6a) Rationale for the control or comparator in the context of the research question, with sources that justify this choice  i) A control group (SA) will be treated with sham acupuncture using Park sham acupuncture needles and non-penetrating sham PTNs  6b) Precise description of the control or comparator. If sham acupuncture or any other type of acupuncture-like control is used, provide details as for items 1) to 3) above  MA: Park sham acupuncture needles and devices will be used in same environment as in the VA group. However, fixed acupoints will be different: a point 1 to 2 cm proximal and 1 cm medial to LI7, a point 1 cm proximal and 1 cm medial to LI11, and a point 1 cm proximal and 1 cm lateral to ST36, each bilaterally  IA: Nonpenetrating sham PTNs, which were designed and validated for blinding for our study, will be used as a control in same environment as in the VA group. The same sized stainless steel rings without needles will be attached to three control points: a point 1 cm proximal and 1 cm medial to LI11, bilaterally, and finger point in the ear contralaterally. |

Abbreviations: STRICTA Standards for Reporting Interventions in Clinical Trials of Acupuncture. MA Manual Acupuncture; IA Intradermal Acupuncture; PTN Press Tack Needle; AD Atopic Dermatitis; VA Verum Acupuncture; SA Sham Acupuncture; KMD Korean Medicine Doctor

**Supplementary table 2. Analysis of the blind test**

| Response | VA group | SA group | p-value |
| --- | --- | --- | --- |
| I think I’m in VA group | 12  (75.0%) | 7  (43.8%) | 0.0719 |
| I think I’m in SA group | 4  (25.0%) | 9  (56.2%) |  |

Values are represented as numbers (%). Data were analyzed using chi-squared test.

**Supplementary table 3. Analysis of the credibility test**

|  | VA (n=18) | SA (n=17) |
| --- | --- | --- |
| Week 0 | 18.0 (1.9) | 17.4 (2.7) |
| Week 4 | 18.2 (2.7) | 18.1 (3.2) |
| Week 8 | 16.8 (4.8) | 17.3 (4.2) |

Values are the sum of four 6-point likert scale question. Mean (SD).

The questions are as follows; 1. Do you expect itching to get better with acupuncture treatment? 2. Would you recommend acupuncture treatment to other atopic dermatitis patients? 3. Does acupuncture treatment seem reasonable? 4. Do you think acupuncture treatment is effective for other diseases?


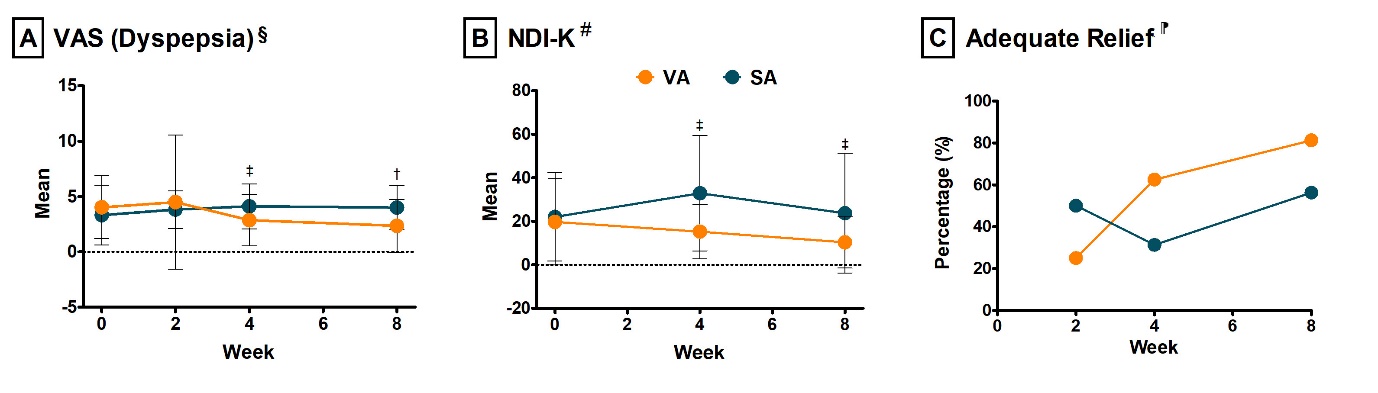
Supplementary fig. 1. Periodic analysis of dyspeptic symptoms

(A, B) Repeated-measure ANOVA was used to see the changes in each variable over time. ANCOVA was used to compare each variable between the two group at weeks 2, 4, and 8, respectively. (C) Trend test was conducted to see the trend of change of AR. The value represents the percent of ‘yes’ responses. ^‡^ p < 0.05, ^†^ p < 0.01
